# Young people who self-harm: a prospective 1-year follow-up study

**DOI:** 10.1007/s00127-015-1149-4

**Published:** 2015-11-25

**Authors:** Madiha Majid, Maria Tadros, George Tadros, Swaran Singh, Matthew R. Broome, Rachel Upthegrove

**Affiliations:** College of Medical and Dental Sciences, University of Birmingham, Edgbaston, Birmingham, B15 2TT UK; Birmingham and Solihull Mental Health Foundation Trust, Birmingham, UK; Warwick University, Coventry, UK; Department of Psychiatry, University of Oxford, Oxford, UK; Oxford Health NHS Foundation Trust, Oxford, UK

**Keywords:** Young people, Self-harm, Repetition, Service provision, Engagement

## Abstract

**Purpose:**

To explore repetition, service provision and service engagement following presentation of young people to emergency services with self-harm.

**Methods:**

969 patients who presented to accident and emergency services after self-harm were followed up prospectively for a period of 1 year. Data on rates, method, clinical history, initial service provision, engagement and repetition (defined as re-presenting to emergency services with further self-harm) were gathered from comprehensive electronic records.

**Results:**

Young people were less likely to repeat self-harm compared to those aged 25 and above. A psychiatric history and a history of childhood trauma were significant predictors of repetition. Young people were more likely to receive self-help as their initial service provision, and less likely to receive acute psychiatric care or a hospital admission. There were no differences in engagement with services between young people and those aged 25 and above.

**Conclusion:**

Younger individuals may be less vulnerable to repetition, and are less likely to represent to services with repeated self-harm. All young people who present with self-harm should be screened for mental illness and asked about childhood trauma. Whilst young people are less likely to be referred to psychiatric services, they do attend when referred. This may indicate missed opportunity for intervention.

## Introduction

Self-harm, defined as bodily harm irrespective of motive and intent encompassing self-poisoning and self-injury, is a significant risk factor for completed suicide and is a growing problem in young people in the UK [1, 2]. Almost half of all suicides have a history of self-harm and this figure rises to two-thirds for younger people [3, 4]. Suicide is the leading cause of death in those aged 34 and under [3, 5]. In addition to self-harm being a major risk factor associated with suicide, repeating self-harm and requiring emergency care after acts of self-harm also place a significant burden on the health economy and acute hospitals [6–8].

In the UK, self-harm is a major healthcare problem [9, 10]. Population studies have identified changes in self-harm trends over time changing in line with national suicide rates [9]. Levels of self-harm have risen in young people and now two-thirds of those who self-harm are aged under 35 [11–14]. The largest rise has been identified in the 15–24 years age group [9, 15, 16]. School-based studies have identified that 7–14 % of 15–16 year olds self-harm with self-cutting being a prominent method [17, 18]. However, those presenting to emergency services more commonly present after episodes of self-poisoning or more severe episodes requiring immediate treatment [19, 20]. There are approximately 220,000 episodes of self-harm presenting to emergency services each year in the UK, and trends here also indicate a rise in younger age groups [9, 10]. Once an individual has presented to an emergency department following self-harm, their risk of suicide is elevated considerably; up to 49 times that of the general population [21]. Moreover, a quarter of suicides required hospital treatment for self-harm in the preceding year [3]. This suggests that there is an opportunity to identify high-risk individuals at this crucial time and setting for appropriate management and intervention. In addition, those who present to emergency services are likely to repeat self-harm, thus elevating their suicide risk further [7, 8]. Repetition often occurs quickly following presentation, with 10 % repeating within a week [7, 8]. Because repetition is so common, information on current clinical management and service provision is required to effectively implement strategies to prevent repetition and suicide [22]. Many cohort studies have only included patients assessed by mental health professionals, leading to a possible selection bias towards the more severe cases and poor outcomes, as within standard liaison psychiatry, not all patients presenting with self-harm are necessarily seen by a mental health professional [13].

Currently, NICE guidelines advise that psychosocial assessments should be organised following presentation to emergency services [23]. The benefits of a psychosocial assessment is a well-researched area; multifactorial care is necessary following self-harm [24], and access to care after discharge is important due to the correlation between suicide risk and reduced level of care [13, 25]. In Birmingham, Rapid Access, Interface and Discharge (RAID) services have been pioneered to enhance psychiatric liaison across all patients presenting to acute hospitals with primary mental health needs, such as self-harm and dementia. The RAID service model uses a multiskilled team that provides comprehensive assessment of a person’s physical and psychological well-being in a general hospital setting. It has been shown to be an effective model in terms of reducing both length of stay and avoiding readmission [26]. Evidence, however, indicates that young people may not seek help in emergency services for self-harm [27], and are less likely to attend appointments with mainstream mental health services [28]. “Discontinuity occurs when services should be at their strongest,” as younger people are less likely to engage with health services [29]. Age-specific interventions may be indicated if presentation and engagement are different between age groups.

Thus, whilst research has focused on trends and risk factors of the self-harm population requiring emergency care as a whole, insufficient evidence exists to evaluate repetition in young people or their care provision and subsequent engagement with services following self-harm [34]. This study thus aimed to examine the presentation, repetition, and service provision in a cohort of young people presenting with self-harm to emergency services in a large urban population.

## Methods

### Study design and setting

This was a prospective cohort study of patients who presented to accident and emergency departments following acts of self-harm and received a psychosocial assessment between 1 September 2012 and 30 November 2012. The accident and emergency departments were based on five hospitals of an NHS trust in Birmingham (Birmingham and Solihull Mental Health Foundation Trust: BSMHFT) where the specialist psychiatric liaison service called RAID exists [26]. RAID clinicians assess ALL patients presenting to emergency services with mental health problems, with the aim of allowing early detection to enable appropriate intervention in the form of treatment and advice. The RAID clinician may be a psychiatrist or a psychiatric liaison nurse. Assessments are recorded in detailed electronic patient notes with a unique and comprehensive service user record that covers across the NHS mental health trust. Patients presenting following self-harm were identified via review of all patients assessed by RAID within the study period, and were followed up prospectively for 1 year after the index episode of presentation with self-harm.

Birmingham is a diverse city and has a large population of over a million people. There are more people in younger age groups as just under half of the population is under 30 (46 %), compared to 38 % for England. In addition, approximately 13 % are aged 65 and over in comparison to 16 % for England [30].

### Data collection

Electronic records of patients who received a psychosocial assessment within the study period were individually screened to identify those who had presented to accident and emergency following self-harm. Patients eligible for inclusion were those aged 16 and above, as RAID does not assess individuals under 16 and service provision for younger individuals is likely to be different. Self-harm was defined as self-inflicted bodily harm irrespective of motive and intent (suicidal or non-suicidal). Those who presented solely with suicidal ideation were not included. RAID records are part of a combined integrated single electronic record for all contact with BSMHFT, the NHS mental health trust providing all psychiatric interventions in the city. Notes are recorded in a standardised format and incorporate the psychosocial assessment following the index episode of self-harm, as well as any other contact the patient had with the mental health trust. Data were coded and entered into a database. Every tenth patient was checked to ensure coded data reflected the raw data.

### Patient data

Sociodemographic factors were recorded and included gender, age, ethnicity, employment status, residence and forensic history. Clinical characteristics were also noted and included the psychiatric history, current psychiatric diagnosis and self-harm history. The psychiatric diagnoses gathered were as recorded by the assessing clinician and classified based on the ICD-10 mental and behavioural disorders. The self-harm history included information on prior presentations to emergency departments after self-harm episodes and self-reported self-harm that did not require emergency care. In addition, a past history of attempted suicide was recorded as elicited by the assessing RAID clinician.

Self-harm details at the index episode were recorded and included the method of self-harm, alcohol involvement in the act and precipitating factors (such as relationship problems, child abuse and substance misuse). The method of self-harm was classed as self-poisoning, self-injury or both. Self-poisoning was defined as administration of a drug in an amount excess to the prescribed or recommended dose, and self-injury was defined as self-inflicted damage to body tissue.

Repetition was defined as those representing to any of the five accident and emergency departments following an act of self-harm after the index episode of presentation with self-harm within the study period. The number of self-harm repetitions within the one-year follow-up period was recorded.

### Service data

The initial management of the patient was recorded from patient notes and was defined as the service outcome. The service outcome included:

- A general hospital admission,
- Acute psychiatric care,
- Community psychiatric care referral,
- Primary care,
- Self-help information or advice,
- Discharged from RAID without any further service input.

Acute psychiatric care included care in an inpatient setting such as admission to a psychiatric unit (formal or informal), respite care or referral to a Home Treatment Team (HTT). Community psychiatric care included a referral to either a Community Mental Health Team (CMHT) or a specialist community psychiatric service, such as alcohol and addiction services. Primary care included discharge to the care of the GP or a primary care psychological service referral. Self-help comprised advice or contact numbers for support services and self-help groups.

Service engagement was measured through attendance, non-attendance or self-discharge from services. Attendance data were available for all the acute psychiatric services, community psychiatric care (CMHT) and general hospital admission.

### Ethics

Ethical approval was obtained from the University of Birmingham Ethics review committee.

### Statistical analysis

Data were analysed using SPSS version 21.0.

Analyses were conducted for the youth (16–24 year olds) and the remainder of the sample (those aged 25 years and above). The age groups were chosen in light of significant focus in recent time on the provision and commissioning of services for young people aged 16–25, and transitional issues for young people, such as disengagement, to adult services. Frequencies for each patient variable were calculated for each group. The proportion repeating self-harm was calculated for each group and a Chi squared analysis was undertaken to determine significant differences. Survival analysis included Kaplan–Meier curves and the log-rank test to assess difference in repetition risk between the two age groups. Cox regression analyses were conducted to identify factors associated with the risk of repetition for each age group and were adjusted for gender, marital status, ethnicity, method of self-harm, psychiatric history and precipitating factors such as drug and alcohol misuse, financial problems and relationship problems.

#### Service outcome

Proportions for each service outcome were calculated for both the 16–24 and 25 and above age groups and a Chi squared analysis was conducted to identify significant differences in service allocation. A multinomial logistic regression analysis was also undertaken to identify factors that were significantly associated with the service outcome. Furthermore, the proportion repeating self-harm in those who received further psychiatric care and those who received no further psychiatric care was calculated and a Chi squared analysis conducted.

#### Service engagement

The proportion attending or not attending a service per age group, and the proportion repeating in attenders and non-attenders were also calculated. Chi squared or Fishers exact test analyses were undertaken to determine significant differences.

## Results

### Study population

RAID assessed a total of 3552 individuals within the 3-month study period. 969 of these individuals presented to accident and emergency departments following a self-harm episode and received a psychosocial assessment, of whom 548 (56.6 %) were female and 421 (43.4 %) were male. The median age was 32 years (IQR 23–44), ranging from 16 to 101. Young people constituted 31 % of the sample (*n* = 309).

### Patient characteristics

A higher proportion of young females presented with self-harm compared to those in the 25 and above age group (68.3 vs 51.1 %, *χ*^2^ = 25.41, *p* < 0.01). In addition, younger individuals were more likely to be single (68.7 vs 59.2 %, *χ*^2^ = 9.03, *p* < 0.01) and from black and minority ethnic groups (33.6 vs 17.5 %, *χ*^2^ = 28.19, *p* < 0.01).

Young people were significantly less likely to be unemployed (43.1 vs 74.7 %, *χ*^2^ = 9.03, *p* < 0.01), living alone or homeless (17.5 vs 41.9 %, *χ*^2^ = 51.17, *p* < 0.01) and have a forensic history (18.4 vs 29.9 %, *χ*^2^ = 12.69, *p* < 0.01). Sociodemographic characteristics are detailed in Table 1.

**Table 1:** Patient characteristics including sociodemographic and clinical characteristics and self-harm history

Variables	All ages	16–24 years	25+ years
	*n* (%)	*n* (%)	*n* (%)
Gender (969)			
Male	421 (43.4)	98 (31.7)	323 (48.9)
Female	548 (56.6)	211 (68.3)	337 (51.1)
Ethnicity (893)			
White	692 (77.5)	184 (66.4)	508 (82.5)
Black and minority ethnic	201 (22.5)	93 (33.6)	108 (17.5)
Marital status (899)			
Single	423 (47.0)	184 (66.4)	239 (38.4)
Married/partner	338 (37.6)	84 (30.3)	254 (40.8)
Widowed/divorced/separated	138 (15.4)	9 (3.2)	239 (20.7)
Employment (800)			
Student	96 (12)	86 (32.2)	10 (1.9)
Employed	191 (23.9)	66 (24.7)	125 (23.5)
Unemployed	513 (64.1)	115 (43.1)	398 (74.7)
Residence (899)			
Homeless	48 (5.3)	9 (3.2)	39 (6.4)
Lives alone	259 (28.8)	41 (14.4)	218 (35.5)
Lives with others	592 (65.9)	235 (82.5)	357 (58.1)
Forensic history (848)	223 (26.3)	49 (18.4)	174 (29.9)
Violence towards others	119 (14.3)	23 (8.6)	96 (16.5)
Psychiatric history (950)	546 (57.5)	139 (46.0)	407 (62.8)
Current psychiatric diagnosis (928)	517 (55.7)	124 (41.9)	393 (62.2)
Mood disorder	298 (32.0)	70 (23.6)	228 (36.1)
Schizophrenia, schizotypal & delusional disorders	59 (6.4)	15 (5.1)	44 (7.0)
Disorders of adult personality and behaviour	46 (5.0)	16 (5.4)	30 (4.7)
Psychoactive substance misuse	60 (6.5)	4 (1.4)	56 (8.9)
Self-harm history			
History of attempted suicide (945)	400 (42.3)	115 (38.2)	285 (44.3)
Previous SH ever (949)	540 (56.9)	170 (56.3)	370 (57.2)
Previous SH in past year (934)	322 (34.5)	113 (37.8)	209 (32.9)
A&E SH presentations in past year (908)	190 (20.9)	60 (20.5)	130 (21.1)
Self-reported SH in past year (911)	178 (19.5)	77 (26.2)	101 (16.4)
Previous SH more than 1 year ago (929)	406 (43.7)	128 (42.8)	278 (44.1)
A&E SH presentations more than 1 year ago (894)	251 (28.1)	69 (24.0)	182 (30.0)
Self-reported SH more than 1 year ago (901)	209 (32.2)	82 (28.3)	127 (20.8)

*A&E* accident and emergency, *SH* self-harm

^a^Number of patient cases with available information varied between variables

Those aged 25 years and above were significantly more likely to have a psychiatric history (62.8 vs 46.0 %, *χ*^2^ = 23.74, *p* < 0.01) and a current psychiatric diagnosis (62.2 vs 41.9 %, *χ*^2^ = 33.64, *p* < 0.01). In contrast, younger individuals were significantly more likely to have a self-reported history of self-harm within the last 12 months (26.2 vs 16.4 %, *χ*^2^ = 12.2, *p* < 0.01) and more than 12 months ago (28.3 vs 20.8 %, *χ*^2^ = 6.19, *p* = 0.01). There were no significant differences between age and other self-harm history variables (detailed in Table 2).

**Table 2:** Method of self-harm and precipitating factors by age group

Variables^a^	All ages	16–24 years	25+ years
	*n* (%)	*n* (%)	*n* (%)
Method of SH (969)			
Self-poisoning	704 (72.7)	236 (76.4)	468 (70.9)
Self-injury	214 (22.0)	59 (19.1)	155 (23.5)
Both self-injury and self-poisoning	51 (5.3)	14 (4.5)	37 (5.6)
Drugs in overdose			
Single drug in overdose (931)	418 (44.9)	144 (48.2)	274 (43.4)
Paracetamol	127 (13.6)	59 (20.3)	68 (11.0)
Opioid analgesic	55 (5.9)	18 (6.2)	37 (6.0)
Antidepressant	51 (5.5)	16 (5.5)	35 (5.6)
Multiple drugs in overdose (931)	297 (31.9)	94 (31.4)	203 (32.1)
Self-injury (969)			
Self-cutting	156 (16.1)	48 (15.5)	108 (16.4)
Other self-injury	109 (11.2)	26 (8.4)	83 (12.6)
Alcohol with SH (966)	334 (34.5)	69 (22.3)	265 (40.1)
Precipitating factors to SH			
Alcohol misuse (957)	289 (30.2)	50 (16.4)	239 (36.6)
Drug misuse (956)	145 (15.2)	47 (15.5)	98 (15.0)
Child abuse (sexual/physical/emotional) (952)	204 (21.4)	80 (26.4)	124 (19.1)
Adult abuse (sexual/physical/emotional) (943)	109 (11.4)	33 (10.9)	76 (11.7)
Bereavement (955)	145(15.2)	30 (9.9)	115 (17.6)
Financial problems (956)	113 (11.8)	20 (6.6)	93 (14.3)
Housing problems (957)	75 (7.8)	16 (5.2)	59 (9.0)
Legal problems (959)	29 (3.0)	7 (2.3)	22 (3.4)
Relationship problems (958)	445 (46.5)	165 (54.1)	280 (42.9)
Physical health problems (960)	278 (29.0)	55 (18.0)	223 (34.1)
Self-harm in response to symptoms (961)	36 (3.7)	11 (3.6)	25 (3.8)

^a^Number of patient cases with available information varied between variables

Self-poisoning was the most common method in both 16–24 years (76.4 %) and 25 years and above (70.9 %) age groups. There was no significant association between age and method of self-harm (*χ*^2^ = 3.17, *p* = 0.21).

#### Repetition

27.8 % (*n* = 269) of individuals repeated self-harm and represented to accident and emergency within the follow-up period. 23.6 % (*n* = 73) of young people compared to 29.7 % (*n* = 196) of those aged 25 years and above represented to accident and emergency with an episode of self-harm. This difference was statistically significant (χ^2^ = 3.87, *p* = 0.05).

##### Age and repetition

**Fig. 1:**
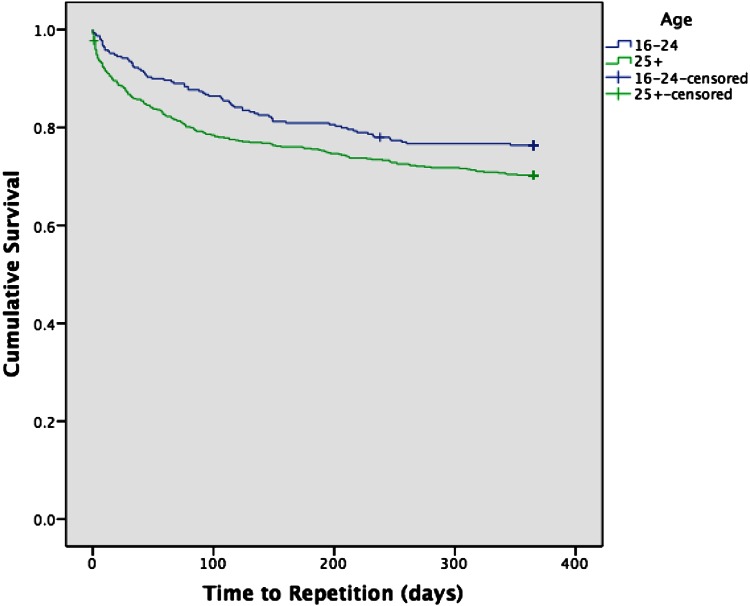
Kaplan–Meier curve showing cumulative probability of self-harm repetition by age groups

Figure 1 illustrates the Kaplan–Meier analysis results. 16–24 year olds had a significantly lower risk of repetition throughout the follow-up period, compared to the 25 years and above group (log-rank test: *χ*^2^ = 4.60, *p* = 0.03). The average time for repetition for 16–24 year olds was 301.40 days (CI 2.87–314.94) and 280.30 days for those aged 25 and above. 39.8 % (*n* = 107) of repetitions occurred within the first 30 days (16–24 years, 20/73, 27.4 %; 25 years and above 87/196, 44.4 %). 81.4 % (*n* = 219) of repetitions had occurred within six months (16–24 years 59/73, 80.8 %; 25 years and above 160/196, 81.6 %).

Cox regression analyses to identify the risk of repetition for both age groups within 12 months of the index episode of self-harm are shown in Table 3. In young individuals, those who had experienced childhood sexual abuse, and had a psychiatric history were at a significantly greater risk of repetition (HR 2.74, CI 1.43–5.25, *p* = <0.01 and HR 2.62, CI 1.46–4.70, *p* = <0.01, respectively). The effect of having a partner or being married significantly reduced the risk of repetition (HR 0.43, CI 0.22–0.83, *p* = 0.01).

**Table 3:** Cox regression analysis results investigating factors associated with self-harm repetition risk by age group

Risk factor	16–24 years	25+ years
	Hazard ratio (95 % CI)	Hazard ratio (95 % CI)
Female gender	1.78 (0.85–3.74)	1.04 (0.76–1.42)
Married or partner	**0.43 (0.22–0.83)**	0.72 (0.52–1.01)
Ethnicity—black and minority ethnic	1.40 (0.78–2.62)	1.09 (0.71–1.67)
Self-cutting involved at index episode	0.99 (0.51–1.91)	**1.58 (1.08–2.29)**
Previous self-harm	1.62 (0.86–3.08)	**2.28 (1.58–3.29)**
Psychiatric history	**2.62 (1.46–4.70)**	**1.66 (1.12–2.45)**
Childhood sexual abuse	**2.74 (1.43–5.25)**	1.39 (0.94–2.05)
Drug misuse	1.86 (0.93–3.72)	1.27 (0.86–1.87)
Alcohol misuse	1.58 (0.80–3.12)	1.20 (0.88–1.65)
Relationship problems	1.04 (0.62–1.76)	0.84 (0.61–1.15)
Financial problems	0.57 (0.13–2.50)	1.09 (0.72–1.67)

Statistically significant hazard ratios are highlighted in bold

In those aged 25 and above, involvement of self-cutting (HR 1.58, CI 1.08–2.29, *p* = 0.02), previous self-harm (HR 2.28, CI 1.58–3.29, *p* = 0.01), and a psychiatric history (HR 1.66, CI 1.12–2.45, *p* = <0.01) significantly increased the risk of repetition.

A cox regression model, adjusted for age, gender, ethnicity and marital status identified that those aged 25 years and above had a significantly higher risk of repetition compared to 16–24 year olds (HR 1.3, CI 1.0–1.8, *p* = 0,05).

#### Service outcome

Young people were significantly more likely to receive self-help as their primary outcome compared to those aged 25 years and above (*χ*^2^ = 5.92, *p* = 0.02). There were no other significant differences between age and other service outcomes as detailed in Table 4.

**Table 4:** Service outcome by age groups

Service outcome	All ages	16–24 years	25+ years	
	*n* (%)	*n* (%)	*n* (%)	
Self-help	112 (11.6)	47 (15.2)	65 (9.8)	*χ* ^2^ = 5.92, *p* = 0.02
General hospital admission	314 (32.4)	92 (29.8)	222 (33.6)	*χ* ^2^ = 1.43, *p* = 0.23
Acute psychiatric services^a^				
Home Treatment Team	100 (10.3)	25 (8.1)	75 (11.4)	*χ* ^2^ = 2.44, *p* = 0.12
Informal admission and respite care	12 (1.5)	2 (0.6)	10 (1.5)	*χ* ^2^ = 1.30, *p* = 0.36
Formal admission	7 (0.7)	3 (1.0)	4 (0.6)	*χ* ^2^ = 0.39, *p* = 0.69
Community services				
Community Mental Health Team	122 (12.6)	41(13.3)	81 (12.3)	*χ* ^2^ = 0.19, *p* = 0.66
Specialist psychiatric services	64 (6.6)	15 (4.9)	49 (7.4)	*χ* ^2^ = 2.25, *p* = 0.13
Primary care services	57 (5.9)	15 (4.9)	42 (6.4)	*χ* ^2^ = 0.87, *p* = 0.35
Discharged from RAID	128 (13.2)	50 (16.2)	78 (11.8)	*χ* ^2^ = 3.50, *p* = 0.07
Other	53 (5.5)	19 (6.1)	34 (5.2)	

^a^Attendance not applicable for formal admission

##### Factors associated with service outcome

A multinomial logistic regression analysis identified that factors significantly associated with service outcome were age, self-harm method, psychiatric diagnosis, marital status, history of suicide, alcohol misuse, and housing problems. Discharge from RAID was used as the reference category. The model accounted for 27.3 % of the variation.

*Self*-*help* Those who were divorced, separated or widowed were 2.5 times more likely to receive self-help (OR 2.52, CI 1.00–6.32, *p* = 0.05).

*General hospital admission* No previous suicide attempt and being aged 16–24 reduced the odds of receiving a general hospital admission by 53 and 48 %, respectively (OR 0.47, CI 0.28–0.77, *p* = 0.03 and OR 0.52, CI 0.31–0.87, *p* = 0.01, respectively).

*Psychiatric care* The odds of receiving a referral for psychiatric care in the community were less likely if there was no history of a suicide attempt (OR 0.46, CI 0.27–0.79, *p* = 0.01). Not having a psychiatric history and no previous suicide attempt also decreased the odds of receiving acute psychiatric care (OR 0.26 CI 0.08–0.85, *p* = 0.03 and OR 0.22, CI 0.12–0.40, *p* = 0<0.01, respectively). Those without housing problems were more likely to receive acute psychiatric care (OR 3.04, CI 1.09–8.52, *p* = 0.03). In addition, those aged 16–24 years were less likely to receive acute psychiatric care (OR 0.47, CI 0.24–0.93, *p* = 0.03).

#### Service outcome and repetition

Those who were admitted to a general hospital or received psychiatric care (primary care, acute or community) were significantly more likely to repeat self-harm compared to those who received no further mental health input (self-help, discharged from RAID and other) (*χ*^2^ = 11.5, *p* < 0.01). This finding remained significant in those aged 25 and above (*χ*^2^ = 8.46, *p* < 0.01) but was not significant for those aged 16–24 (*χ*^2^ = 2.12, *p* = 0.15).

#### Service engagement

In total, there were 122 community psychiatric services (CMHT) referrals, 110 acute psychiatric service referrals or admissions, and 314 general hospital admissions for which attendance data were available. There were no significant differences between age and attendance for acute psychiatric services, CMHT and general hospital admissions, as detailed in Table 5.

**Table 5:** Service engagement by age group

Service	All ages	16–24 years	25+ years	
	*n* (%)	*n* (%)	*n* (%)	
General hospital admission				
Attended	281 (89.5)	82 (89.1)	199 (89.6)	*χ* ^2^ = 0.18, *p* = 0.89
Self-discharge	33 (10.5)	10 (10.9)	23 (10.4)	
Acute psychiatric services				
Attended	104 (94.5)	26 (100)	78 (92.9)	*χ* ^2^ = 1.96, *p* = 0.33
Did not attend	6 (5.5)	0 (0.0)	6 (7.1)	
Community psychiatric services				
Attended	97 (84.3)	31 (81.6)	66 (85.7)	*χ* ^2^ = 0.33, *p* = 0.57
Did not attend	18 (15.7)	7 (18.4)	11 (14.3)	

##### Service engagement and repetition

There was no significant difference in the proportion repeating self-harm in those who attended (32.4 %, *n* = 156) and did not attend services (29.8 %, *n* = 17) following the index episode of self-harm episode (*χ*^2^ = 0.15, *p* = 0.70).

## Discussion

This study identified that young people may be less likely to repeat self-harm after first presentation to emergency services with self-harm. Factors that increased the likelihood of young people repeating included a psychiatric history and a history of child abuse. Young people were more likely to be given self-help information after the presentation with self-harm as the primary outcome. Those who were referred to psychiatric care engaged with services as reflected through high levels of attendance; however, attendance at follow-up appointments did not appear to influence repetition. We also reported that younger individuals were more likely to be from black and minority ethnic (BME) groups than the over 25 age group; this may reflect population demographics which show a significant young BME population in Birmingham. Whilst BME groups may be less likely to self-harm or seek help despite increased incidences of mental illness [31, 32], this may not reflect a young BME sample and there is evidence that some younger BME groups, such as Asian women, are at increased risk of self-harm [33].

### Repetition

Our findings that young individuals are less vulnerable to repeat self-harm and represent to emergency services are in contrast to reports of young people being more vulnerable to repetition [34]. It is possible that young people do repeat self-harm but do not attend accident and emergency services a second time; this may be a likely explanation for the apparent lower repetition observed by this group. However, our results still indicate that a high proportion of individuals of all ages repeat self-harm and attend accident and emergency services in the first 12 months. This emphasises the need to identify high-risk individuals and direct them to appropriate services. The overall repetition rate of 28 % in our sample is higher than figures from previous studies but in line with those exploring repetition in adolescents, and may also reflect the RAID service where all presentations are assessed by this enhanced liaison service [35, 36].

Findings from our study show that those who were at increased risk of repetition were those who had a psychiatric history, for both the youth and those aged 25 and over. In those aged 25 and above, self-cutting and previous self-harm increased the risk of repetition. These predictors of repetition should remain an integral part of assessment following self-harm [34, 37]. The role of cutting as a method has also been implicated with an increased risk of fatal repetition [35]. For young people, a history of childhood sexual abuse was a significant predictor in addition to a psychiatric history. This highlights the importance of the link between childhood abuse, particularly sexual abuse, with repetition and suicide attempts due to its long-term psychological impact [38, 39]. Thus, if these results are translated into clinical practice, the self-harm presentation may provide an opportunity to detect risk factors at hospital that may otherwise remain undetected, and clinicians should enquire about childhood abuse with every young person who presents with self-harm.

Research has previously shown that repetition risk increases with age, concurring with our findings, and is higher in middle-aged adults than in older adults [39]. Acts of self-harm by older adults are also more fatal and have different motivations [35, 40]. This further illustrates that age-specific psychosocial assessments may be needed following self-harm to meet the needs of high-risk patients [41].

### Service outcome and engagement

Younger individuals were more likely to receive self-help information and this may reflect the stigma that younger individuals associate with mental health services [42]. Alternatively, perceived stigma on the assessing practitioner’s part may leave them less likely to refer to services; further research could investigate whether age impacts the service provided based on the assessing clinicians views. However, it should be considered that as with any service provided, medical severity and risk assessments will have an influence on the appropriate initial management. Younger individuals were also associated with reduced odds of receiving a general hospital admission and acute psychiatric care. This may be a reflection of the reduced severity of the self-harm act, and is evident from the literature that indicates younger people are more likely to act impulsively [38]. Birmingham, as any large UK city, has a growing range of non-statutory providers of lower level psychological interventions, such as ‘Open Door’ youth counselling, primary care psychological services, public health initiatives and self-help via websites (for example, http://youthspace.me) [43]. Furthermore, factors that were associated with being referred to psychiatric care were a history of a suicide attempt and psychiatric history, reflecting that high-risk patients are being directed to appropriate services. This further indicates that management for young people could be appropriate.

However, contact with services did not seem to reduce the proportion repeating in those receiving psychiatric care and those who were referred to psychiatric services were more likely to repeat self-harm. This may illustrate a ‘selection effect’ where high-risk cases are being directed towards psychiatric services [44, 45]. We suggest that rather than showing a negative effect of psychiatric care, individuals referred are highly likely to repeat self-harm and are thus being appropriately managed in secondary psychiatric services rather than primary care. Interventions that can be better placed to reduce repetition within psychiatric care are indeed a challenge, particularly in relation to disorders with high levels of repeated self-harm.

Evidence shows that following acute psychiatric admission after self-harm, the risk of repeating is high and suggests that interventions should be in place to prevent this [46]. The National Institute of Clinical Excellence recommends targeted psychological treatments for the long-term management of self-harm and future research should investigate whether young people who present with self-harm are appropriately accessing these interventions [47]. Therapies need to be explored with different age groups to identify those that are most effective in reducing repetition. Transitional issues between child and adolescent mental health services to adult services have been highlighted as contributory to reduced engagement with services in young individuals. However, previous studies have also shown that engagement after self-harm specifically is not problematic [48], particularly in urban areas where follow-up is high, especially in those with suicidal intent [49].

### Strengths and limitations

The strengths of this study include the impact of age on repetition and service provision in a large sample size and the inclusion of all patients presenting with self-harm. It is also the first study to consider both outcome and attendance to services, adding a unique perspective to the literature. However, this study is not without limitations. There are three main limitations specific to this study. Firstly, results are to be interpreted in the context of the methodological limitations; additional markers of socioeconomic status such as occupation and education were not recorded and were, therefore, not included in the analyses. Secondly, it was not possible to capture repeated self-harm that did not result in emergency attendance, and did not have access to data from primary care follow-up. Thirdly, only the initial service outcome was considered as some patients may have received more than one service provision, for example those who were admitted to a general hospital may have received further psychiatric input. The number of patients who were referred for further psychiatric care may, therefore, be underrepresented. Finally, suicide as an outcome was not recorded to allow comment on the risk of repetition on age and mortality in this sample; a much larger sample size would be required for the study to have a high statistical power.

### Conclusion

Repetition is common following presentation to emergency services with self-harm. Age differences are apparent where younger individuals may be less vulnerable to repetition, whilst older age groups may be more vulnerable as a result of longer on-going psychiatric illness and repeated self-harm. Younger adults with a psychiatric history and presence of childhood sexual abuse are most likely to represent, whereas in the over 25’s repeated self-harm, cutting and a psychiatric history are most at risk of further presentation. This highlights the importance of a comprehensive assessment to identify factors that put individuals most at risk of repetition which will allow care to be provided for secondary prevention of self-harm. Furthermore, age is likely to have a role in the service provided where young people are more likely to receive self-help, and less likely to be admitted to hospital. However, age is not the sole determinant of service provision and consideration of other factors, such as past suicidal attempt, is involved in determining the care received. There were no age-related differences in service engagement and this may reflect the referral of complex cases to the appropriate service in keeping with the severity of self-harm. This does highlight the need to evaluate the effectiveness of services provided, particularly for high-risk patients in attempting to prevent future events of self-harm. Whilst young people are less likely to be referred to psychiatric services following self-harm, they do attend when referred and this may indicate missed opportunity for more effective interventions.
